# Supplementary material for: Quantitative SUMO proteomics identifies PIAS1 substrates involved in cell migration and motility
Source: Nat Commun. 2020 Feb 11;11:834. doi: 10.1038/s41467-020-14581-w (PMC7012886; doi:10.1038/s41467-020-14581-w)
Supplement: Supplementary file 1 — Supplementary Information [file 41467_2020_14581_MOESM1_ESM.pdf]

Quantitative SUMO proteomics identifies PIAS1 substrates involved in cell migration and motility

Chongyang Li[1,2], Francis P. McManus[1], Cédric Plutoni[1], Cristina Mirela Pascariu[1], Trent Nelson[1,2], Lara Elis Alberici Delsin[1,3], Gregory Emery[1,3], Pierre Thibault [1,2,4,5*]

[1]Institute for Research in Immunology and Cancer, [2]Department of Molecular Biology, [3]Department of Pathology and Cell Biology, [4]Department of Chemistry, [5]Department of Biochemistry, Université de Montréal, Québec, Canada

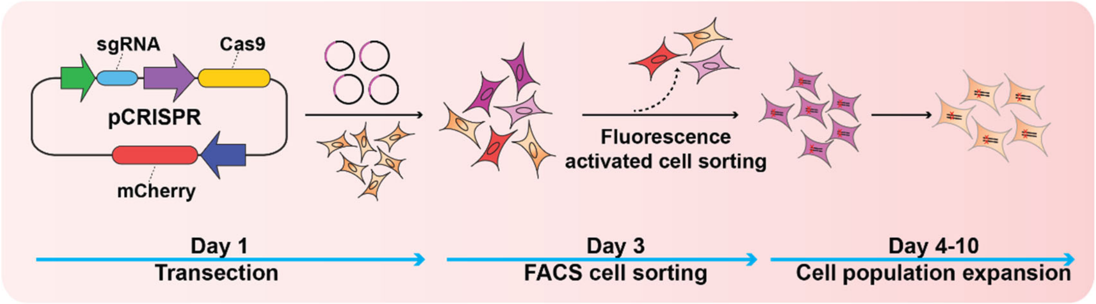

Supplementary Figure 1: Overview of PIAS gene knockout by CRISPR/Cas9-based gene editing technology.

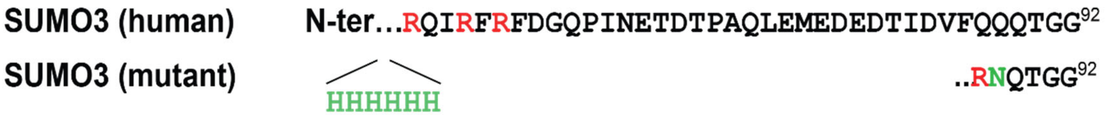

Supplementary Figure 2: Protein sequences of the endogenous SUMO3 and SUMO3m.

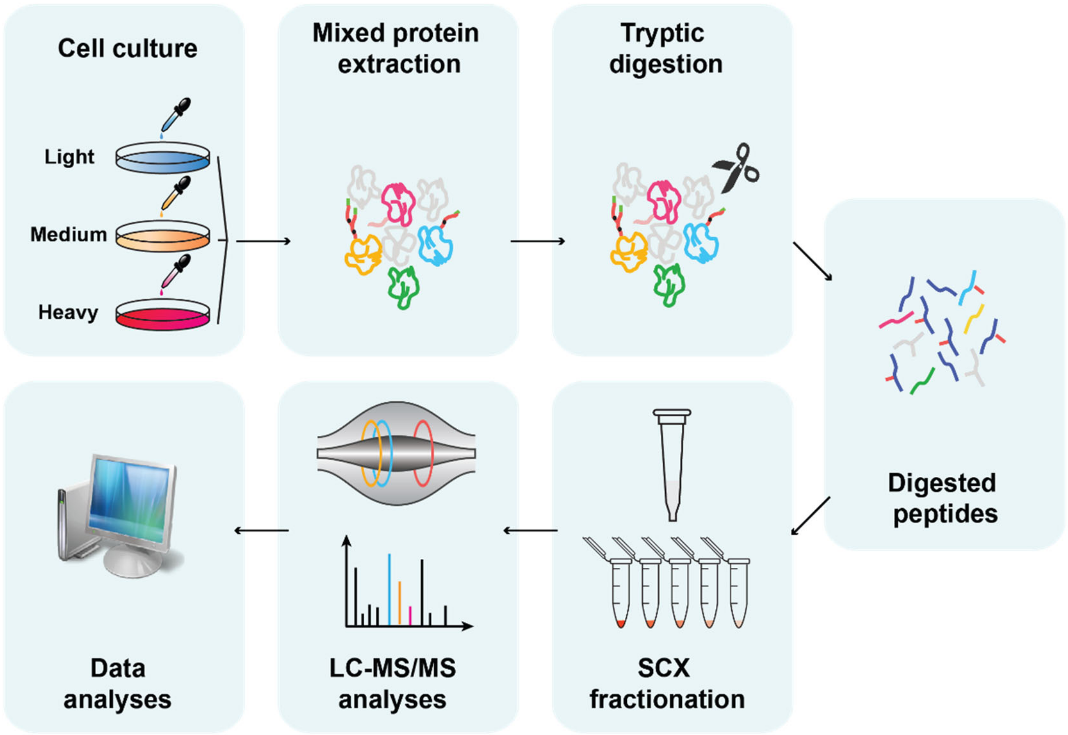

Supplementary Figure 3: Overview of proteome identification. SILAC labelled cells were lysed and combined in a 1:1:1 ratio based on protein content. Mixed cell lysates were digested using trypsin with a ratio trypsin: protein = 1:50. After desalting and drying, tryptic peptides were fractionated on SCX columns and injected on a Tribrid Fusion mass spectrometer. Peptide identification and quantification was performed using MaxQuant.

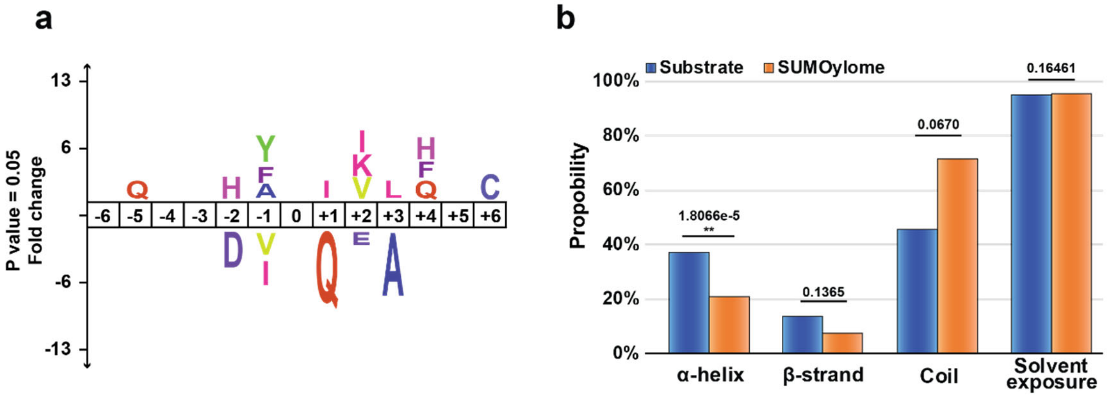

Supplementary Figure 4: Structural analysis of identified substrates. (a) Icelogo of the amino acid sequence surrounding the PIAS regulated SUMO sites compared to the whole SUMO proteome. (b) Secondary structure prediction of identified PIAS1 substrates vs identified SUMOylome, **p<0.01, Student's t-test.

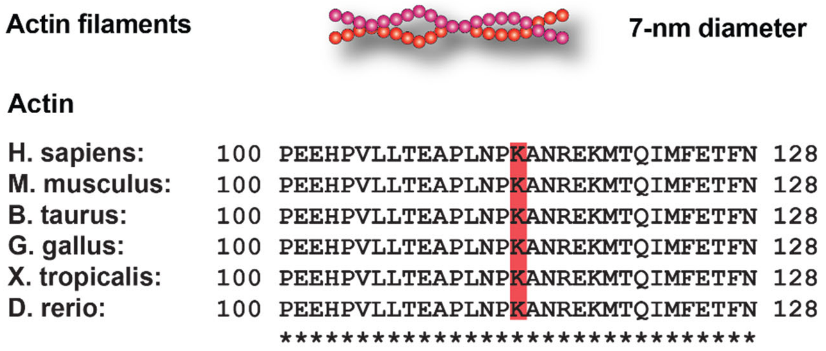

Supplementary Figure 5. Cartoon representation of the identified SUMOylation sites on Actin at Lys 115. Protein sequence alignment of Actin across six different species showing that Lys 115 is highly conserved.

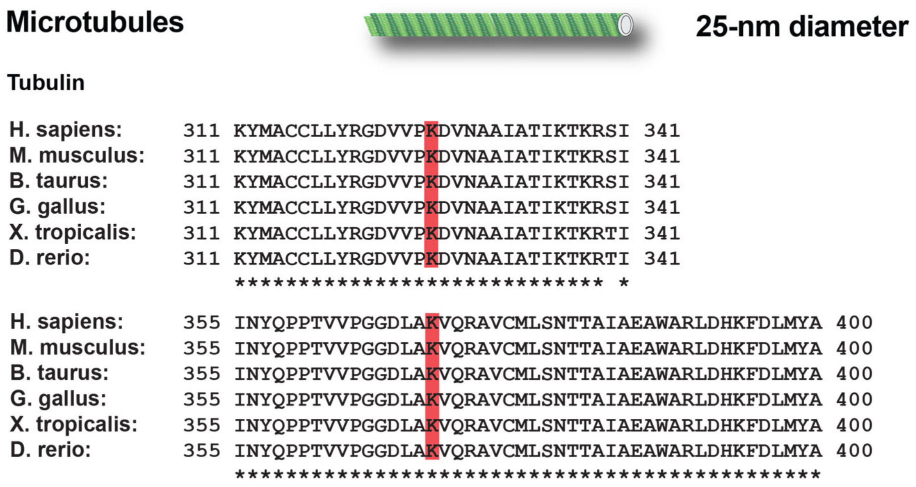

Supplementary Figure 6. Cartoon representation of the identified SUMOylation sites on Tubulin at Lys 326 and Lys 370. Protein sequence alignment of Tubulin across six different species showing that all these lysines are highly conserved.

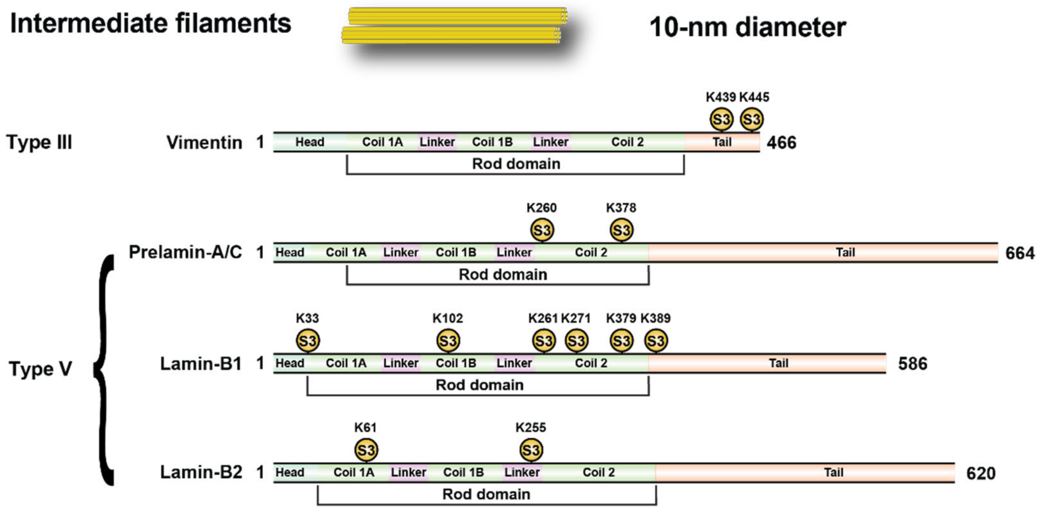

Supplementary Figure 7. Cartoon representation of the identified SUMOylation sites on different intermediate filament proteins

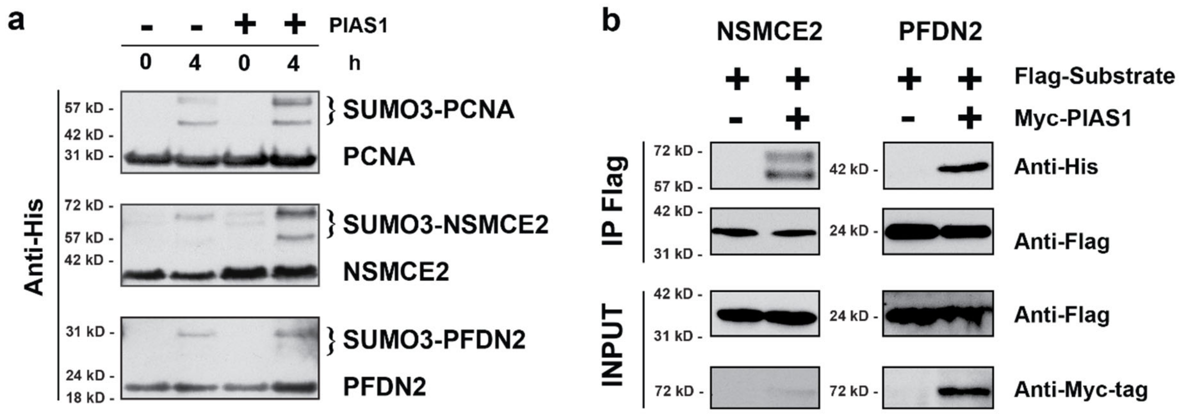

Supplementary Figure 8. Validation of SUMOylation on identified PIAS1 substrates. (a) In vitro SUMOylation assay was performed with or without PIAS1 in a buffer containing SAE1/SAE2, UBC9, SUMO3, ATP and substrates. The samples were incubated at 37°C for 4h and examined by western blot. In vitro SUMO assays show that PIAS1 enhances SUMOylation of PCNA, NSMCE2 and PFDN2. (b) HEK293 SUMO3m cells were co-transfected with the indicated vectors (top), immunoprecipitated with an anti-Flag antibody, and examined by western blot. SUMOylation of NSMCE2 and PFDN2 were also enhanced by PIAS1 in cells.

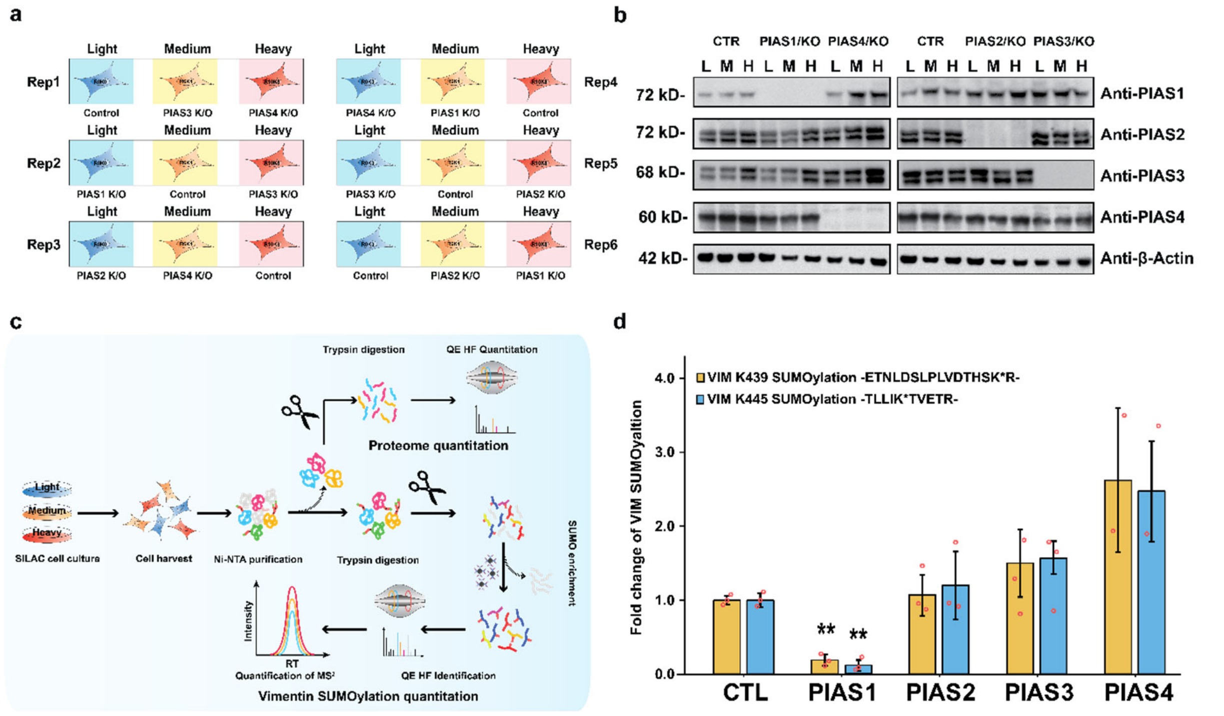

Supplementary Figure 9. Workflow for the quantification of vimentin SUMOylation following knockout of different PIAS E3 SUMO ligases. a) SILAC labeling strategy for each individual sample replicates. The strategy ensures that each cell line is in all three SILAC channels to eliminate bias caused by a given channel. b) Endogenous PIAS protein expression in SILAC-labeled HEK293 PIAS-knockout cells were analyzed by western blot. The blot reveals that SILAC channels for each cell line express similar levels of PIAS and that the knockout for each cell line is specific to the respective isoforms. c) Experimental workflow for the quantification of VIM SUMOylation by mass spectrometry. SUMOylated proteins were first enriched by Ni-NTA, digested on beads with trypsin and modified tryptic peptides were purified by SUMO remnant immunoaffinity purification prior to targeted LC-MS/MS analyses to quantify changes in vimentin SUMOylation. Targeted LC-MS/MS analyses were performed on a Q-Exactive HF mass spectrometer with an inclusion list to acquired MS/MS spectra of the $[M+3H]^{3+}$ precursor ions of the isotopically labeled SUMOylated peptides ETNLDSLPLVDTHSK*R and TLLIK*TVETR where * indicates SUMOylation site. We also analyzed by LC-MS/MS in data-dependent acquisition the tryptic peptides from the flow through proteins to normalize protein abundance across the 6 different samples. d) Fold change of VIM SUMOylation relative to control cells based on SILAC ratios and sample normalisation. VIM SUMOylation is largely abolished at K439 and K445 following CRISPR/Cas9 PIAS1 KO. n=3 biologically independent samples. Data represent the mean ± S.D., error bars represent S.D., **$p<0.01$, Student's t-test).

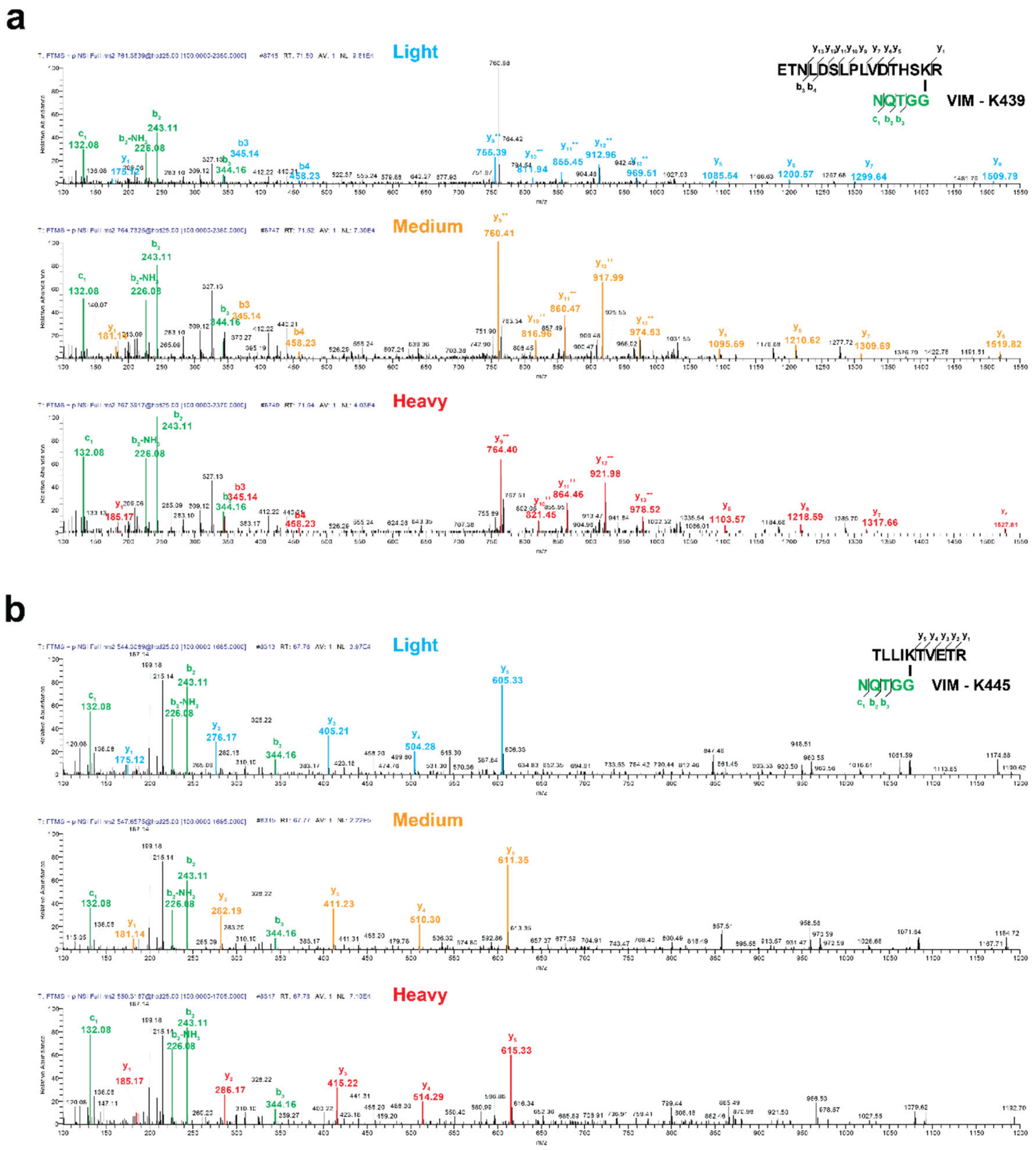

Supplementary Figure 10. Representative MS/MS spectra of SUMOylated vimentin peptides a) ETNLDSLPLVDTHSK*R and b) TLLIK*TVETR in each SILAC channel, where * designates SUMOylation site. Spectra from each SILAC channel correspond well with the other channels.

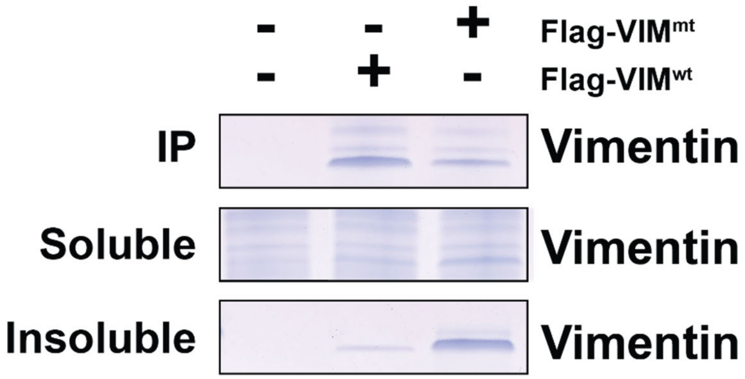

Supplementary Figure 11. SDS-PAGE gel fraction of Flag-VIM^{wt}, Flag-VIM^{mt} and negative control from immunoprecipitation, soluble fraction and insoluble fraction used for LC-MS/MS analysis

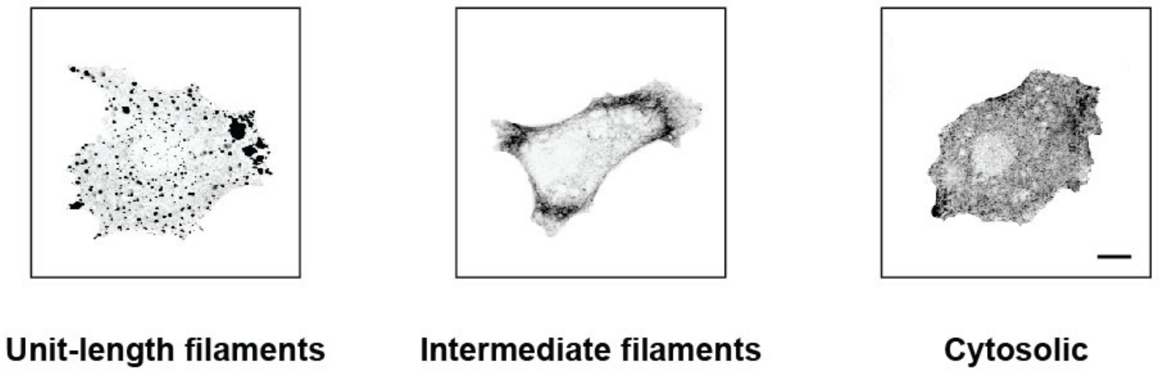

Supplementary Figure 12. Representative depiction of different forms of vimentin in MCF-7 cells. Scale bar: 10 µm.

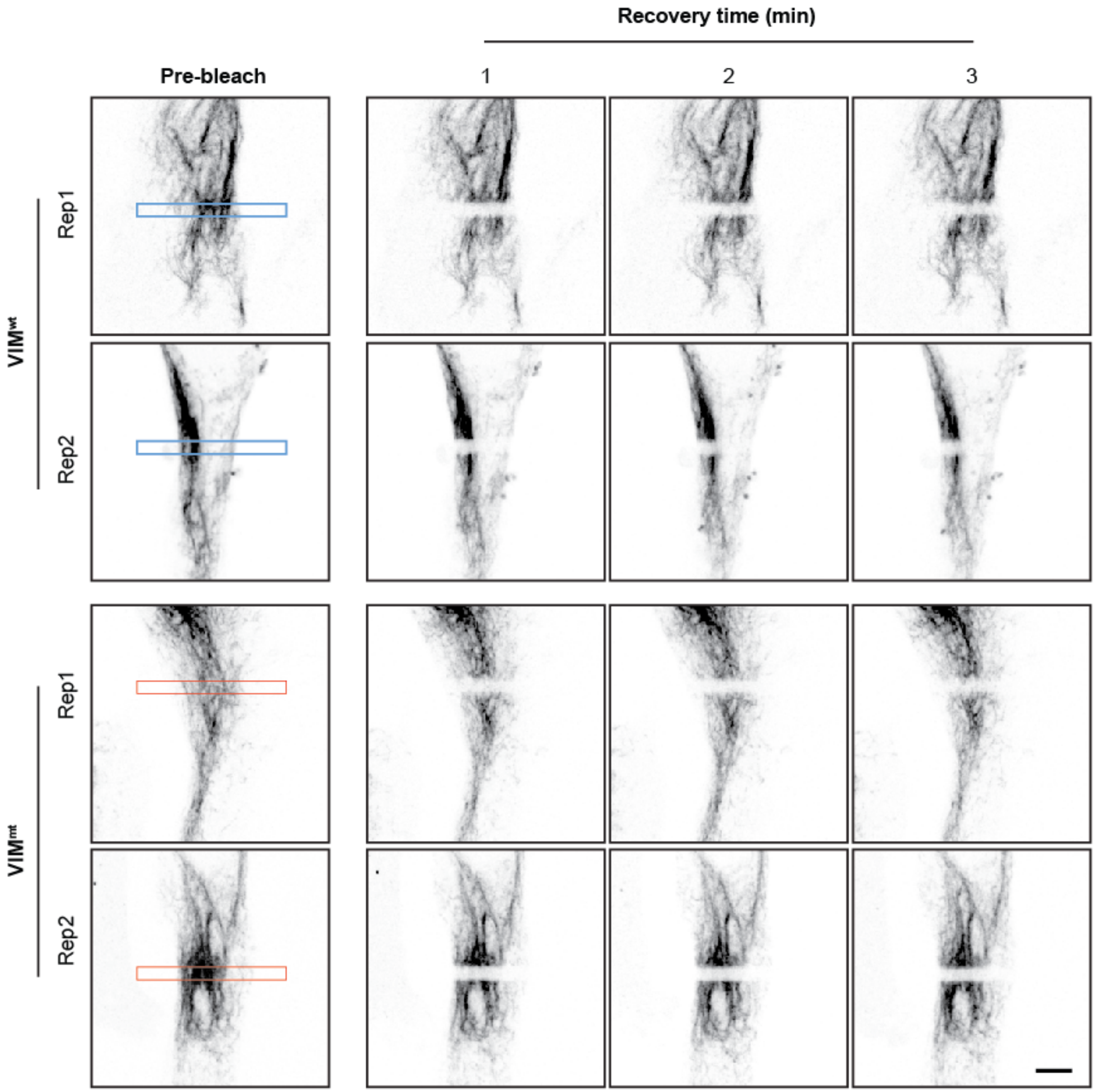

Supplementary Figure 13. FRAP assays of Emerald-VIM^{wt} and VIM^{mt} in MCF-7 cells. Selected images of fluorescence recovery after bleaching are shown. Scale bar: 5 µm.
