# Supplementary material for: Quantitative SUMO proteomics identifies PIAS1 substrates involved in cell migration and motility
Source: Nat Commun. 2020 Feb 11;11:834. doi: 10.1038/s41467-020-14581-w (PMC7012886; doi:10.1038/s41467-020-14581-w)
Supplement: Supplementary file 3 — Description of Additional Supplementary Files [file 41467_2020_14581_MOESM3_ESM.docx]

**Description of Supplementary Files**

**File Name: Supplementary Data 1**

**Description:** List of quantified proteome from large‐scale SILAC quantitative proteomics analysis.

**File Name: Supplementary Data 2**

**Description:** List of quantified SUMOylated peptides from large‐scale SILAC quantitative SUMO proteomics analysis.

**File Name: Supplementary Data 3**

**Description:** List of quantified phosphorylation sites on soluble and insoluble VIM.
