# Supplementary material for: Quantitative SUMO proteomics identifies PIAS1 substrates involved in cell migration and motility
Source: Nat Commun. 2020 Feb 11;11:834. doi: 10.1038/s41467-020-14581-w (PMC7012886; doi:10.1038/s41467-020-14581-w)
Supplement: Supplementary file 7 — Reporting Summary [file 41467_2020_14581_MOESM7_ESM.pdf]

nature research

Corresponding author(s): Pierre Thibault

Last updated by author(s): Dec 10, 2018

Reporting Summary

Nature Research wishes to improve the reproducibility of the work that we publish. This form provides structure for consistency and transparency in reporting. For further information on Nature Research policies, see Authors & Referees and the Editorial Policy Checklist.

Statistics

For all statistical analyses, confirm that the following items are present in the figure legend, table legend, main text, or Methods section.

n/a	Confirmed	
☐	☒	The exact sample size (n) for each experimental group/condition, given as a discrete number and unit of measurement
☐	☒	A statement on whether measurements were taken from distinct samples or whether the same sample was measured repeatedly
☐	☒	The statistical test(s) used AND whether they are one- or two-sided<br>*Only common tests should be described solely by name; describe more complex techniques in the Methods section.*
☒	☐	A description of all covariates tested
☒	☐	A description of any assumptions or corrections, such as tests of normality and adjustment for multiple comparisons
☒	☐	A full description of the statistical parameters including central tendency (e.g. means) or other basic estimates (e.g. regression coefficient) AND variation (e.g. standard deviation) or associated estimates of uncertainty (e.g. confidence intervals)
☒	☐	For null hypothesis testing, the test statistic (e.g. F, t, r) with confidence intervals, effect sizes, degrees of freedom and P value noted<br>*Give P values as exact values whenever suitable.*
☒	☐	For Bayesian analysis, information on the choice of priors and Markov chain Monte Carlo settings
☒	☐	For hierarchical and complex designs, identification of the appropriate level for tests and full reporting of outcomes
☒	☐	Estimates of effect sizes (e.g. Cohen's d, Pearson's r), indicating how they were calculated

Our web collection on statistics for biologists contains articles on many of the points above.

Software and code

Policy information about availability of computer code

Data collection	MS data were acquired on an Orbitrap Fusion Mass spectrometer (Thermo Fisher Scientific) using the manufacturer acquisition software Xcalibur.
Data analysis	MS data were analyzed using MaxQuant (version 1.5.3.8). MS/MS spectra were searched against UniProt/SwissProt database (http://www.uniprot.org/) . Additional details are provided in On line methods

For manuscripts utilizing custom algorithms or software that are central to the research but not yet described in published literature, software must be made available to editors/reviewers. We strongly encourage code deposition in a community repository (e.g. GitHub). See the Nature Research guidelines for submitting code & software for further information.

Data

Policy information about availability of data

All manuscripts must include a data availability statement. This statement should provide the following information, where applicable:

- Accession codes, unique identifiers, or web links for publicly available datasets
- A list of figures that have associated raw data
- A description of any restrictions on data availability

The mass spectrometry proteomics data have been deposited to the ProteomeXchange Consortium (http://proteomecentral.proteomexchange.org) via the PRIDE partner repository with the dataset identifier <PXD011932>. Reviewer account details: Username: reviewer91455@ebi.ac.uk, Password: ScCPBsuC

Field-specific reporting

Please select the one below that is the best fit for your research. If you are not sure, read the appropriate sections before making your selection.

☒ Life sciences ☐ Behavioural & social sciences ☐ Ecological, evolutionary & environmental sciences

For a reference copy of the document with all sections, see nature.com/documents/nr-reporting-summary-flat.pdf

Life sciences study design

All studies must disclose on these points even when the disclosure is negative.

Sample size	For cell proliferation, cell migration and cell invasion assays:<br>Cell numbers were counted by adding equal parts of 0.4% trypan blue (Invitrogen ref T10282) dye to the cell suspension to obtain a 1 to 2 dilution (example: 20 µl of cells to 20 µl of trypan blue). 10 µl cell suspension was filled into a cell counting chamber slide (Invitrogen ref C10228) and counted under a Countess™ Automated Cell Counter(Invitrogen ref C10227). For each sample, measurements were performed 3 times. The initial cell numbers are 1000 cells/well for cell proliferation assay, 320000 cells/well for cell migration assay and 30000 cells/well for cell invasion assay.<br>For the large scale proteomic experiments:<br>Three triple SILAC experiments were conducted with a control sample and 2 samples transfected with PIAS1 expressing vectors creating 6 treated samples vs 3 control samples. For each SILAC replicate, 5.33 mg of total cell extract (TCE) was used for each channel.<br>Immunoprecipitation of VIM in tandem with LC-MS experiments were conducted once.<br>FRAP assays were conducted in biological duplicates.
Data exclusions	No data were excluded from any analysis.
Replication	All experiments were repeated for a total of 3 times. This includes, sample processing for the large scale proteomic studies, cell proliferation assay, cell migration assay and cell invasion assay.
Randomization	Large scale proteomic studies were conducted using reversed labelling, where the labelling state of the control sample was swapped in every biological replicates (ie: REP1 controls is light, REP2 control is medium and REP3 control is heavy).
Blinding	No blinding was necessary.

Reporting for specific materials, systems and methods

We require information from authors about some types of materials, experimental systems and methods used in many studies. Here, indicate whether each material, system or method listed is relevant to your study. If you are not sure if a list item applies to your research, read the appropriate section before selecting a response.

Materials & experimental systems

n/a	Involved in the study
☐	☒ Antibodies
☐	☒ Eukaryotic cell lines
☒	☐ Palaeontology
☒	☐ Animals and other organisms
☒	☐ Human research participants
☒	☐ Clinical data

Methods

n/a	Involved in the study
☒	☐ ChIP-seq
☒	☐ Flow cytometry
☒	☐ MRI-based neuroimaging

Antibodies

Antibodies used	Cell Signaling Technology (CST):<br>PIAS1 (D33A7) XP® Rabbit mAb #3550, α-Tubulin (11H10) Rabbit, Myc-Tag (71D10) Rabbit mAb #2278 mAb, β-Actin (13E5), Rabbit mAb #4970, Histone H3 (D1H2) XP® Rabbit mAb #4499, Anti-rabbit IgG (H+L), F(ab')2 Fragment (Alexa Fluor® 555 Conjugate) #4413S, Anti-rabbit IgG, HRP-linked Antibody #7074S, Anti-mouse IgG, HRP-linked Antibody #7076S<br>Sigma-Aldrich:<br>A2220 ANTI-FLAG® M2 Affinity Gel, F7425 Sigma ANTI-FLAG® antibody produced in rabbit.<br>Santa Cruz Biotechnology:<br>PML Antibody (H-238): sc-5621 produced in rabbit.<br>Qiagen:<br>34660-Penta·His Antibody, BSA-free (100 µg) produced in mouse.
Validation	All antibodies were purchased and not produced in house, except for anti-K(NQTGG). All antibodies produced bands at the desired molecular weight. The anti-K(NQTGG) was characterized extensively by our previous proteomic studies, where peptides

bearing K(NQTGG) epitope were enriched after the IP as detected by LC-MS/MS (PMID: 28098164, PMID: 29048423 and PMID: 25391492).

Eukaryotic cell lines

Policy information about cell lines

Cell line source(s)	HEK293 SUMO3m, HeLa and MCF-7.
Authentication	The cell lines were not authenticated.
Mycoplasma contamination	All cell lines were tested for mycoplasma and were negative
Commonly misidentified lines (See ICLAC register)	*Name any commonly misidentified cell lines used in the study and provide a rationale for their use.*
